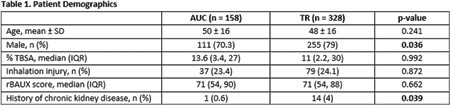# 6 A Multicenter Outcome Analysis of Vancomycin Dosing Strategies in Burn Patients

**DOI:** 10.1093/jbcr/irae036.006

**Published:** 2024-04-17

**Authors:** Allison N Boyd, David M Hill, Richard M Santos

**Affiliations:** Eskenazi Health, Zionsville, IN; Regional One Health, Memphis, TN; Eskenazi Health, Zionsville, IN; Regional One Health, Memphis, TN; Eskenazi Health, Zionsville, IN; Regional One Health, Memphis, TN

## Abstract

**Introduction:**

Current guidelines support an area under the curve (AUC)-based dosing strategy for vancomycin. Previous literature evaluating dosing in burn patients consistently reports that higher doses and/or more frequent intervals are required in patients with thermal injury. However, optimal empiric dosing recommendations and monitoring are not well defined and burn patients are often excluded from studies driving guideline recommendations. The objective of this study was to evaluate both AUC and non-AUC-guided dosing strategies in a multicenter analysis. The primary hypothesis was that therapeutic drug monitoring via AUC-guided dosing would result in 25% higher clinical success compared to trough-based dosing (TR) in patients with burn or inhalation injury.

**Methods:**

This was a multicenter, retrospective study including 14 burn centers. Patients admitted to one of the study sites from 1/1/17 through 8/31/22 with a cutaneous burn or inhalation injury who received intravenous vancomycin for at least 48 hours were included. Patients were excluded if they lacked at least one steady state trough concentration, experienced an acute kidney injury within 24 hours of vancomycin initiation, were treated for an infection with a known vancomycin MIC greater than or equal to 2, or received continuous infusion vancomycin. Patients were evaluated for clinical success and grouped according to method of monitoring: AUC vs. TR. Clinical success was defined as a composite of absence of 5 criteria: 1) AKI 2) lack of resolution 3) relapse 4) therapy change or 5) death.

**Results:**

A total of 486 patients receiving 517 courses of vancomycin were included. Select patient demographics are included in Table 1. Primary and secondary outcomes are included in Table 2. There was no difference in clinical success between dosing strategies. This remained true after controlling for center, burn severity, and primary infection source. There was no difference in target attainment. The TR group had significantly higher rates of AKI, with concomitant piperacillin/tazobactam remaining significant after multivariate regression analysis.

**Conclusions:**

No differences in clinical success were seen between AUC vs. TR dosing strategies and both groups had high attainment of therapeutic concentrations. AUC-guided dosing may reduce rates of AKI in burn patients with a history of kidney disease, kidney injury on admission, or when vancomycin is being administered with concomitant nephrotoxic medications.

**Applicability of Research to Practice:**

To our knowledge, this is the first multicenter study to evaluate vancomycin dosing strategies in patients with thermal or inhalation injury.